# Age-Dependent Risks of COVID-19 Putatively Caused by Variant Alpha in Japan

**DOI:** 10.3389/fpubh.2022.837970

**Published:** 2022-06-10

**Authors:** Taishi Kayano, Katsuma Hayashi, Tetsuro Kobayashi, Hiroshi Nishiura

**Affiliations:** School of Public Health and Graduate School of Medicine, Kyoto University, Kyoto, Japan

**Keywords:** coronavirus, statistical estimation, variant of concern, mutation, severity

## Abstract

**Background:**

Osaka, the third largest prefecture in Japan, experienced a rapid replacement of preexisting strains of severe acute respiratory syndrome coronavirus 2 (SARS-CoV-2) by variant alpha during March-April 2021. Assessing the burden of variant alpha on health centers and medical institutions is vital to anticipating the surge of patients. The present study aimed to estimate the age-dependent risks of coronavirus disease (COVID-19) putatively caused by variant alpha in Japan, focusing on epidemiological dynamics in Osaka.

**Methods:**

Descriptive analyses were conducted using data on confirmed, severe and fatal cases of COVID-19 from 16 November 2020 to 22 May 2021. All cases were divided into 6–9 age groups to compare the risks of confirmed diagnosis, severe illness and death from COVID-19 with variant alpha to those caused by preexisting strains.

**Results:**

Individuals with COVID-19 aged under 30 years were more likely to be infected with variant alpha than those in their 40s. The incidence of severe illness and death among all age groups with COVID-19 due to variant alpha was higher than that due to preexisting strains. Patients older than 40 years experienced an increased risk of severe illness and death if infected with variant alpha. However, the proportion of severe cases was lower in the group aged 80 years and older infected with variant alpha than in those infected with preexisting strains.

**Conclusion:**

Our analysis suggests that the incidence of infection among young people aged below 30 years old increased relative to ordinary strains. Risks of severe illness and death in patients with variant alpha COVID-19 was higher than in those with preexisting strains in Osaka, Japan. However, a decrease in the risk of severe illness was observed in people aged ≥80 years, which is probably because medical facilities in Osaka were overwhelmed in April and May 2021. Continuous monitoring of COVID-19 cases with new variants is vital to secure sufficient medical resources for all patients who require medical care.

## Introduction

The emergence of novel severe acute respiratory syndrome coronavirus 2 (SARS-CoV-2) variants, especially those that are categorized as variants of concern (VOC), has elevated tensions not only in countries affected by a surge in the number of patients but also in countries starting the rollout of vaccination programs. Because the efficacy and effectiveness of vaccines against SARS-CoV-2 could potentially vary between variants and vaccines, researchers have been still assessing them carefully. Examining the influence of VOC on the risks of infection, severe disease and death is critical because preventing the collapse of healthcare facilities is key to confronting the outbreak of SARS-CoV-2 that has been seen in many countries (1–3).

Coronavirus disease (COVID-19) is caused by acute infection of the respiratory tract with SARS-CoV-2 leading to symptoms including cough, fever and fatigue (4). Although approximately 80% of confirmed cases of COVID-19 are characterized by mild symptoms or are asymptomatic, approximately 20% of confirmed cases are diagnosed with severe and critical disease that requires admission to an intensive care unit (ICU) and/or care unit with a medical ventilator for a few weeks (5). Similar to other infectious disease, the risk of severe disease and mortality in patients with COVID-19 varies across age groups (6, 7). Elderly people are more vulnerable to COVID-19 than younger people, and many patients diagnosed with severe disease could occupy hospital beds for a few weeks. Moreover, a published study suggested that unfair allocation of medical resources can limit the capacity of healthcare facilities, resulting in increased risks of severe illness and death due to COVID-19 (8). Thus, preventing the collapse of healthcare facilities and maintaining basic operation must be achieved during the pandemic.

After the SARS-CoV-2 lineage B.1.1.7 with the N501Y mutation, also known as variant alpha, was confirmed in the UK in September 2020, that variant rapidly spread across the world and started replacing preexisting strains in many countries, including Japan (9–11). Variant alpha appeared to be approximately 1.5 times more transmissible than preexisting SARS-CoV-2 variants (12–14). In addition, some studies suggested that the variant could cause more severe illness than preexisting SARS-CoV-2 variants (15, 16). Thus, there were countries where more intensive non-pharmaceutical interventions (NPIs), such as national lockdowns and curfews, were implemented to suppress a wave of cases with a VOC (17, 18).

**Figure 1:**
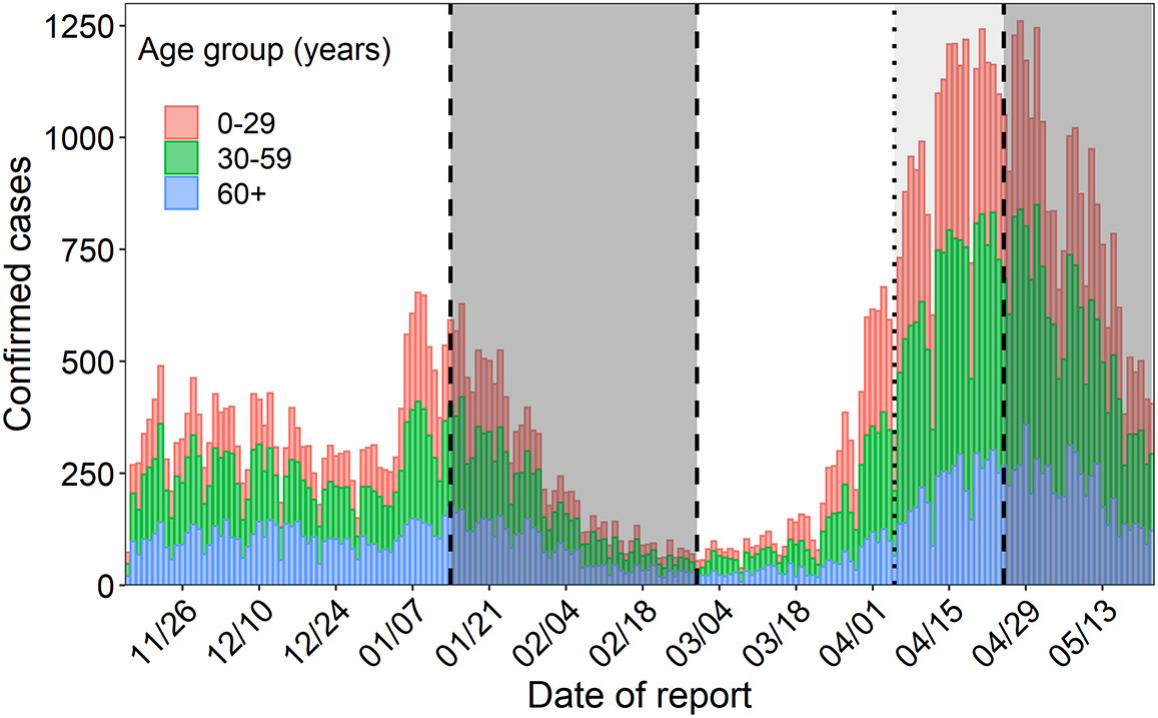
Incidence over time in Osaka. The colors of the bars represent age group. The gray shaded area signifies the state of emergency period and the area shaded in light gray represents the pre-emergency measures period.

The first COVID-19 cases with variant alpha in Japan were recognized in late December 2020 (19). Since then, the situation of the spread of variant alpha was only understood by screening for the VOC with N501Y mutation among confirmed polymerase chain reaction (PCR)-positive cases in Japan. Although the government started screening for cases with VOCs across the whole country with a target of screening 40% of PCR-positive samples, those values depended on the screening capacity of each prefecture (11, 20). The number of confirmed cases of COVID-19 reached 650 in the third wave in early January 2021 and peaked at more than 1,200 in the fourth wave in the middle of April 2021 in Osaka, the second largest metropolitan area following Tokyo in Japan (Figure 1). Osaka experienced a rapid replacement of preexisting strains and an increase in the proportion of cases of variant alpha among PCR-positive samples in screening tests earlier than other prefectures (21). According to Osaka prefecture, the weekly proportion of samples positive for the VOC with N501Y mutation (mostly variant alpha) among PCR-positive samples was estimated to be more than 40% since early March, and subsequently reached more than 65% at the end of March 2021 (22).

Assessing the burden of variant alpha on health centers and medical institutions is essential to anticipating the surge of patients in terms of the necessary beds, personnel and personal protective equipment. Therefore, risks of infection, severe disease and death due to variant alpha should be quantified using the Japanese dataset of confirmed cases of COVID-19. The present study estimated the age-dependent risks of COVID-19 putatively caused by variant alpha in Japan using epidemiological data from Osaka. This assessment provides not only an understanding of risks of variant alpha in Japan but also an insight into COVID-19 caused by other VOCs in the future.

## Materials and Methods

### Epidemiological Data

The patients' data were publicly available and extracted from the website of Osaka prefecture (23). The data of confirmed cases, severe cases and deaths were available from 16 November 2020; however, to minimize the influence of intensive NPIs during the states of emergency, the periods of the second (from 14 January 2021 to 28 February 2021) and third (from 25 April 2021 to the end of May 2021) states of emergency were excluded from the analysis (Figure 1).

Although Osaka prefecture requested and issued pre-emergency measures preceding the state of emergency, those interventions targeted restaurants and bars serving foods and drinks (24). The same requests were continuously applied during the non-pre-emergency period, i.e., March 2021, especially in Osaka city; therefore, we decided not to use the data from during the state of emergency in Osaka.

**Figure 2:**
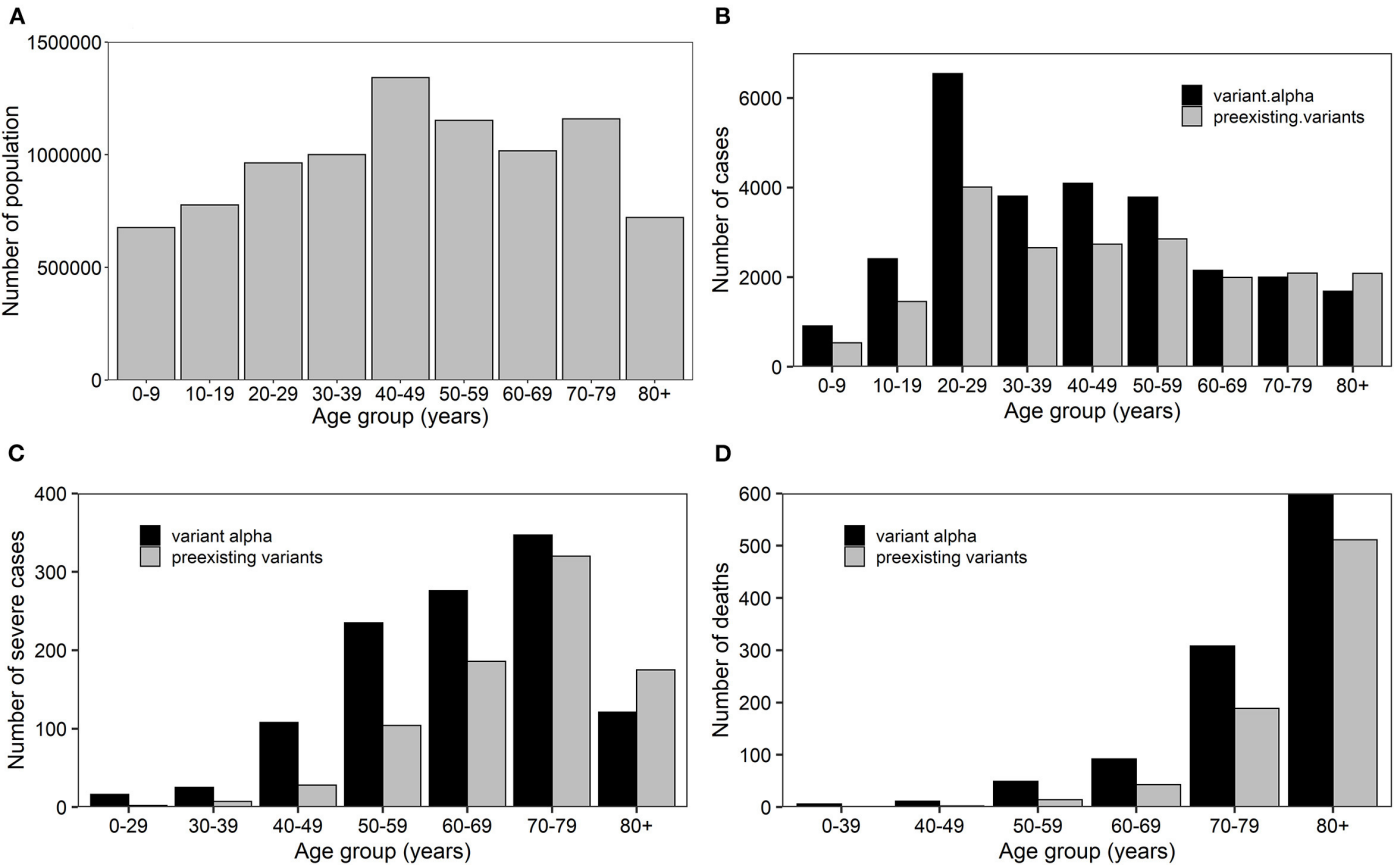
Age distribution of epidemiological characteristics of COVID-19 in Osaka. **(A)** Population dynamics in Osaka in 2019. **(B)** Absolute number of cases, **(C)** severe cases and **(D)** deaths due to preexisting and variant alpha strains of COVID-19 in Osaka.

Because the proportion of screenings positive for COVID-19 with variant alpha exceeded 50% after March 2021, we assumed that all confirmed cases of COVID-19 were due to variant alpha in Osaka from March. Therefore, for our estimates, the confirmed cases from 16 November 2020 to 13 February 2021 and cases from 1 March 2021 to 24 April were considered to be due to preexisting strains and variant alpha, respectively. Confirmed cases were divided into nine age groups, i.e., 0–9, 10–19, 20–29, 30–39, 40–49, 50–59, 60–69, 70–79, and ≥80 years old. Accounting for the delay between infection and report, severe cases of COVID-19 with non-alpha and alpha strains were used for the analysis from 16 November 2020 to 27 January 2021 and from 15 March 2021 to 8 May 2021, respectively. Severe cases were divided into seven age groups, i.e., 0–29, 30–39, 40–49, 50–59, 60–69, 70–79, and ≥80 years old due to the scarcity of cases in the younger population. Confirmed deaths that reported the date of death were used for the analysis from 16 November 2020 to 10 February and from 29 March 2021 to 22 May for non-alpha and alpha strains, respectively, considering the delay from infection to death. The deaths were divided into six age groups, i.e., 0–39, 40–49, 50–59, 60–69, 70–79, and ≥80 years old due to the scarcity of the deaths in those aged 0–39 years. These three different variables are shown in Figure 2 by age group.

### Statistical Model

#### Confirmed Cases

To make the numbers of each period comparable, the confirmed cases, severe cases and deaths were converted from raw numbers (i.e., observed absolute number) to rates (i.e., number over 30 days).

For the analysis of confirmed cases, the odds ratios (OR) of age group *a* with variant alpha to cases with non-alpha variants were calculated using a group aged 40–49 years as the reference as shown below.


$$\begin{array}{l} OR_{\alpha ,\;a}=\frac{c_{\alpha ,\;a}}{c_{\bar{\alpha }}}/\frac{c_{\alpha ,\;40s}}{c_{\bar{\alpha },\;40s}} \end{array}$$

where *c*_α, *a*_ represents the confirmed cases with the variant alpha in age group *a* and $\bar{\alpha }$ represents the confirmed cases with the non-alpha variants in age group *a*.

#### Severe Cases and Deaths

To analyze severe cases and deaths due to variant alpha and preexisting strains of COVID-19, three different measures were calculated. First, the odds ratio of COVID-19 cases in age group *a* with alpha for severe cases and deaths were calculated as


$$\begin{array}{l} OR_{\alpha ,\;a}=\frac{s_{\alpha ,\;a}}{c_{\alpha ,\;a}}/\frac{s_{\bar{\alpha },\;a}}{c_{\bar{\alpha },\;a}} \end{array}$$

where *s* represents the total number of severe cases or deaths. Secondly, we calculated the proportion of severe cases or deaths in confirmed cases with variant alpha or with preexisting strains to explore the vulnerable age groups for those risks. Finally, the severe case rate ratio and death rate ratio of COVID-19 cases in age group *a* with variant alpha or with non-alpha variants were calculated and compared to the reference age group of 40–49 years using the equation:


$$\begin{array}{l} Rate\;ratio_{\alpha ,\;a}=\frac{s_{\alpha ,\;a}}{N_{a}}/\frac{s_{\alpha ,\;40s}}{N_{40s}},\\ Rate\;ratio_{\bar{\alpha },\;a}=\frac{s_{\bar{\alpha },\;a}}{N_{a}}/\frac{s_{\bar{\alpha },\;40s}}{N_{40s}}. \end{array}$$

where *N*_*a*_ represents the population of age group *a* in 2019, which is shown in Figure 2A. Rate ratio is used in epidemiology to compare incidence (rate) between two different subgroups of certain events at a given time. Therefore, to explore two different age groups by different variants of COVID-19, rate ratios were calculated with respect to each outcome, becoming severe illness and death. All calculations were conducted using R (version 4.0.5). The odds ratios and their 95% confidence intervals (CIs) were calculated using the “Epi” package, which was based on the unconditional maximum likelihood estimation for estimated values and the Wald method for CIs (25). Other estimates were calculated according to the abovementioned equations and CIs were estimated using the Wald method for rate ratios and Wilson score interval for proportions.

## Results

Table 1 shows the odds ratios of putative cases of variant alpha COVID-19 among different age groups relative to the reference age group of 40–49 years. Variant alpha was more likely to be detected in infected individuals <30 years old than in those aged 40–49 years, although the 95% CIs crossed unity (1.0). In contrast, those aged 50 years old and older were less likely to have variant alpha COVID-19 than those aged 40–49 without crossing unity for 95% CIs. Therefore, the risk of infection with variant alpha COVID-19 tended to be higher among young people than among those aged 40–49 years. Additionally, that risk was smaller among people aged 50 years old and older than those aged 40–49 years.

**Table 1:** Odds ratios of putative cases of variant alpha COVID-19 by age group.

**Age group (years)**	**Incidence (30 days)**		**OR (95% CI)**	***p*** **-value**
	**Alpha**	**Preexisting**		
0–9	496.4	273.1	1.13 (0.96, 1.33)	0.14
10–19	1315.1	739.3	1.11 (0.99, 1.24)	0.08
20–29	3568.9	2040	1.09 (1.00, 1.19)	0.06
30–39	2078.2	1352	0.96 (0.87, 1.05)	0.37
40–49	2234.7	1389.7	1	-
50–59	2065.6	1452.2	0.88 (0.80, 0.97)	<0.05
60–69	1172.2	1014.9	0.72 (0.65, 0.80)	<0.05
70–79	1089.8	1062.2	0.64 (0.57, 0.71)	<0.05
≥80	919.1	1060.2	0.54 (0.48, 0.60)	<0.05

Table 2 shows the odds ratios for severe cases of and deaths due to variant alpha COVID-19 relative to preexisting strains by age group. Severe illness and death were higher among all age groups in those with variant alpha COVID-19 than in those with preexisting strains, although the 95% CIs crossed unity among patients aged <40 years and >79 years old with respect to severe cases and among patients aged <50 years with respect to deaths. Focusing on ORs with < 0.05 *p*-value, 3.18 among patients aged 40–49 with respect to severe cases and 3.89 among patients aged 50–59 with respect to deaths were the highest values, respectively.

**Table 2:** Odds ratios of severe cases and deaths due to variant alpha COVID-19 by age group.

	**Severe cases**		**Deaths**	
**Age group (years)**	**OR (95% CI)**	***p*** **-value**	**OR (95% CI)**	***p*** **-value**
0–29	6.02 (0.63, 57.83)	0.11	5.60 (0.17, 187.34)	0.34
30–39	3.08 (0.86, 11.02)	0.08		
40–49	3.18 (1.69, 6.01)	<0.05	5.41 (0.45, 65.45)	0.26
50–59	2.11 (1.48, 3.00)	<0.05	3.89 (1.47, 10.28)	<0.05
60–69	1.71 (1.28, 2.27)	<0.05	2.93 (1.63, 5.26)	<0.05
70–79	1.40 (1.11, 1.78)	<0.05	2.51 (1.86, 3.39)	<0.05
≥80	1.06 (0.75, 1.50)	0.79	2.44 (2.00, 2.98)	<0.05

**Figure 3:**
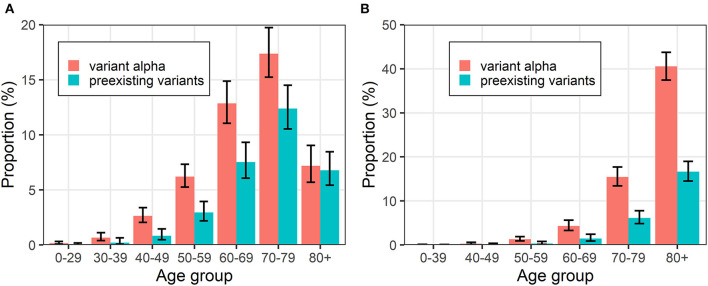
Proportions of events in putative cases of with variant alpha and preexisting strain COVID-19 by age group. **(A)** Severe cases and **(B)** deaths are shown. Error bars represent 95% confidence intervals.

Figure 3 shows the proportion of severe cases and deaths in confirmed cases with variant alpha and preexisting strains. Regarding severity, while the proportion of severe cases increased as groups grew older up to 79 years old, the values were lower among those aged ≥80 years because relevant patients appeared to die without satisfying the definition of severe disease due to the limited ICU/ventilator capacity, especially in April in Osaka. For deaths, the trends for both types of variants were similar, and the risk of death increased with increasing age. This is because death is a measure which is more likely to be counted without biases compared to severe cases. According to Figure 3, the age group trends are the same as both variants.

**Figure 4:**
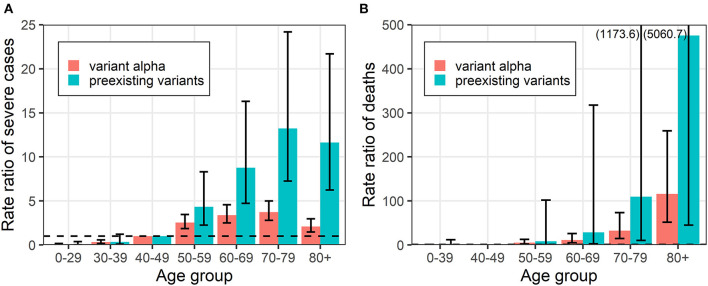
Rate ratios of events due to variant alpha and preexisting strain COVID-19 by age group. **(A)** Severe cases and **(B)** deaths are shown. Error bars represent 95% confidence intervals. Values in parentheses represents the upper bound of the 95% confidence interval.

Figure 4 shows the rate ratio for severe cases and deaths due to variant alpha and preexisting strains by age group relative to the reference age group of 40–49 years. Patients older than the reference age group had increased risk of severe illness and death. The reason that the rate ratios of severe cases decreased in the group aged ≥80 years is the same as that mentioned above, i.e., the limited hospital capacity in Osaka. It is notable that the difference in risk between age groups considerably narrowed, indicating that risk increased in almost all age groups with variant alpha. In other words, elderly people are not necessarily the only key-target group that is crucial to consider as demands of hospital beds for patients with variant alpha.

## Discussion

The present study explored the risks of infection, severe illness and death due to infection with variant alpha COVID-19 relative to those due to preexisting strains by age group in Osaka, Japan. Our analysis found that the risk of severity increased in all age groups, especially in those aged 0–29 years although the confidence interval crossed unity. Furthermore, the trend for the risk of death was similar to the risk for severe illness. These results coincide with those of earlier studies exploring variant alpha COVID-19 in some countries. In Denmark, for example, the risk of hospital admission of COVID-19 cases with variant alpha was increased in all age groups compared to that of the cases with preexisting variants (26). While there is research that a risk of deaths among the cases with variant alpha decreased in some European countries and European Economic Area by the analysis considering the age effect (though the confidence intervals crossed unity and the risk of hospitalizations increased), the risk increased in all studied age groups in the UK (27, 28). It was challenging to find a declined risk of severe illness in older age groups in other countries; however, the reduction in the risk in patients aged ≥80 years indicates that there was a possibility that those cases died not meeting the definition of severe illness due to the limited number of beds at medical institutes in Osaka. Considering this situation, we cannot deny that the increased risk of death stemmed from overwhelmed healthcare facilities. The vaccine rollout for healthcare workers started on 17 February 2021 in Japan; subsequently, the vaccination campaigns for elderly people, which has been led by local governments, started in the middle of April 2021. According to the vaccination record system in Japan, the vaccination coverage among people aged ≥ 65 years was < 5% by the middle of May 2021 in Osaka; therefore, the effect of vaccination on the result can be ignored (29). In addition, Osaka prefecture officially announced that the first case with the delta variant was confirmed on 14 May 2021 in Osaka; therefore, we believe that our analysis excluded the influence of the spread of that variant (30).

Our study has a few “take home” messages. First, the risk of infection with variant alpha was higher in younger age groups relative to patients aged 40–49 years while the risk in other age groups was lower. Differential contact behaviors among school children and minors may play an important role in the differences in age-dependent transmission dynamics. The increase in variant alpha incidence overlapped with the end and start periods of the academic year, i.e., March and April in Japan, during which adolescents frequently organize farewell and welcome events, possibly fueling a series of transmission. Second, the risk of severe illness and death increased in all age groups with variant alpha. Looking at the proportions of those outcomes, elderly people were still the most vulnerable group. The abrupt decline in the proportion and rate ratio of cases of severe illness in people aged ≥80 years suggests that hospitals were overwhelmed by the surge of cases of variant alpha COVID-19 in March and April in 2021 in Osaka. If there were sufficient ICU and medical ventilation/extracorporeal membrane oxygenation (ECMO) for all patients with COVID-19, the number of severe cases would have increased like the number of deaths. However, our results explicitly reflected that the situation in Osaka was grave at that time, and many elderly people died without intensive care. Third, relative to cases in people aged 40–49 years, the death/severe illness rate ratios were higher in elderly groups. The risks in people aged 40–49 years increased with variant alpha; therefore, the risk in elderly people relatively decreased. This means that the disparity in risk between age groups shrank with variant alpha. Thus, both elderly patients and those of working age are at increased risk of severe illness and death following infection with variant alpha.

A few limitations must be acknowledged in the present study. First, the abrupt surge in patients infected with COVID-19 in the fourth wave led to the “hospital overload” in Osaka. Although that influenced the outcome of care/treatment of patients, especially with respect to deaths, we could not exclude the burden on the healthcare system from the analysis. Thus, the result regarding severity may reflect effects of not only of variant alpha but also overwhelmed hospitals. Second, it is obvious that the variant of COVID-19 in each case should be classified using real-time PCR if the influence between variants is explored. However, the confirmation of cases with variant alpha was implemented using screening tests among confirmed cases with COVID-19, and the individual data were not publicly available in Osaka. It was impossible to match cases; therefore, we decided to distinguish cases between preexisting and variant alpha by period. Removing the influences of strong NPIs in Osaka, analyses periods for the confirmed cases with preexisting variants and with variant alpha were 90 days and 55 days, respectively. Two declarations of the state of emergency and the emergence of variant delta imposed on such inconsistent study periods, although our analyses were conducted by converting the incidences (rates). Indeed, the definition of cases with either variant in the present study has considerable uncertainty as it was based on inference by period of VOC prevalence in the study area and not confirmed by VOC determination by individual PCR test for variant Alpha or whole genome sequencing of SARS-CoV-2 RNA. However, considering the speed of the spread of variant alpha in Osaka and other countries, such as the UK, we believe it was a reasonable and the best attempt to address the limitations of the data source (21, 22). Third, unfortunately, the data regarding cases with severe illness and deaths were only available from November 2020 in Osaka; therefore, we tried to consider the cases with as little bias as possible. Although the number of available cases for the analysis were reduced, datasets from during the state of emergency were excluded to avoid the effect of strong NPIs. The cases reported under the pre-emergency measures were used in the analysis because those interventions appeared to target the shops providing foods and alcohol and had less effect on the number of COVID-19 cases (24, 31).

Despite the abovementioned limitations, the publicly available data regarding confirmed cases, severe cases and deaths in Osaka allowed us to compare the risks between preexisting strains and variant alpha COVID-19 by age group using simple calculations. Considering the expansion of the vaccination campaign targeting elderly people and healthcare workers, severe cases are expected to be predominantly seen in people aged 40–64 years. In addition, the delta variant, which appears to have higher transmissibility and possibly more likely to cause severe illness, will become an additional threat in Japan (32, 33). In conclusion, our analysis suggests that the risks of severe illness and deaths in patients with variant alpha COVID-19 was higher than that in patients with preexisting strains in Osaka, Japan. Moreover, a reduction in the risk of severe illness was observed in people aged ≥80 years, which can probably be attributed to overwhelmed medical facilities in Osaka in April and May 2021. Thus, continuous monitoring of COVID-19 cases with variants is necessary for the national and local governments to secure sufficient medical resources for all patients who require medical care.

## Data Availability Statement

Publicly available datasets were analyzed in this study. The datasets analyzed for this study can be found in the Osaka prefectural government website (https://www.pref.osaka.lg.jp/iryo/2019ncov/).

## Author Contributions

HN conceived the study idea. TK analyzed the empirical datasets. TK and HN reviewed the results and drafted manuscript together. All authors gave comments and approved the final version of the manuscript.

## Funding

This study was supported by funding from Health and Labor Sciences Research Grants (19HB1001, 19HA1003, 20CA2024, 20HA2007, and 21HB1002 to HN), the Japan Agency for Medical Research and Development (JP20fk0108140 and JP21fk0108612 to HN), the Japan Society for the Promotion of Science KAKENHI (TK: 21K10495 and HN: 17H04701 and 21H03198), the Inamori Foundation, the GAP Fund Program of Kyoto University, the Japan Science and Technology Agency CREST program (JPMJCR1413 to HN), and the SICORP program (JPMJSC20U3 and JPMJSC2105 to HN). The funders played no role in the study design, data collection and analysis, decision to publish, or preparation of the manuscript.

## Conflict of Interest

The authors declare that the research was conducted in the absence of any commercial or financial relationships that could be construed as a potential conflict of interest.

## Publisher's Note

All claims expressed in this article are solely those of the authors and do not necessarily represent those of their affiliated organizations, or those of the publisher, the editors and the reviewers. Any product that may be evaluated in this article, or claim that may be made by its manufacturer, is not guaranteed or endorsed by the publisher.
